# Flash laser annealing for controlling size and shape of magnetic alloy nanoparticles

**DOI:** 10.3762/bjnano.1.7

**Published:** 2010-11-22

**Authors:** Damien Alloyeau, Christian Ricolleau, Cyril Langlois, Yann Le Bouar, Annick Loiseau

**Affiliations:** 1 Matériaux et Phénomènes Quantiques, UMR 7162, Bâtiment Condorcet, Case 7021, Université Paris 7 / CNRS, 75205 Paris Cedex 13, France, Phone : +33 1 57 27 69 83; 2 Laboratoire d’Etude des Microstructures – Unité mixte ONERA / CNRS, BP 72, 92322 Châtillon Cedex, France

**Keywords:** magnetic alloy nanoparticles, nanoparticle morphology, nanosecond pulsed laser annealing, order-disorder transformation

## Abstract

We propose an original route to prepare magnetic alloy nanoparticles with uniform size and shape by using nanosecond annealing under pulsed laser irradiation. As demonstrated here on CoPt nanoparticles, flash laser annealing gives an unprecedented opportunity to control the size and the shape of bimetallic nanoparticles without changing their composition. The mechanisms involved in the complete reshaping of the nanoparticle thin films are discussed and it is also shown that order-disorder phase transformations occur under laser irradiation. This technique is then very interesting for magnetic alloy nanoparticles studies and applications because it opens up a new way to fabricate size-controlled spherical nanoparticles with narrow size dispersion.

## Introduction

Future high-density recording systems require 10 nm magnetic grains with a high magnetic anisotropy (K_u_) to insure their thermal stability [1]. CoPt and FePt nanoparticles (NPs) in the chemically ordered L1_0_ structure [2] are very promising materials for such magnetic applications, because of their 10 times larger K_u_ as well as the larger saturation magnetization compared to CoCr-based alloys used nowadays in the recording systems [3–5]. However the understanding of their size-dependant properties and their future applications depend on the ability to synthesize NPs with a very good control over the size distribution and the chemical composition. Up to now, only chemical synthesis is able to produce monodisperse CoPt [6] and FePt [7–8] NPs with a polydispersity (that is, standard deviation divided by the mean size) as small as 10%. However, chemically prepared monodisperse bimetallic NPs are limited to sub-10 nm diameter, and postsynthesis thermal treatments used to increase NPs size, inevitably widen size dispersion. Vapour phase homogenous nucleation allows the fabrication of sub-10 nm and sup-10 nm particles, but their polydispersity varies from 30% to 60% with cluster size. This broad size distribution makes the study of the structural and magnetic properties of NPs complicated, because it drastically influences the results of commonly used analysis techniques, such as X-ray diffraction and SQUID. For that reason, single NP analysis techniques are necessary to understand size effects on the NP properties [9–11].

In this article, we propose an original route to prepare CoPt bimetallic NPs with uniform size and shape by using nanosecond annealing with pulsed laser radiation at 248 nm. This technique has already been successfully applied to Ag [12–14] and Au [15–20] NPs in solution, or on a substrate, by using laser energy in the UV range or at the plasmon resonant wavelength of the metal. We will show here that similar results can be obtained on bimetallic NPs by using a nanosecond pulsed laser beam, without changing the NPs composition. These developments open up a new way to design magnetic alloys NPs with ideal morphologies and size for magnetic studies and applications.

## Results and Discussion

CoPt NP thin films on amorphous alumina (a-Al_2_O_3_) were produced by pulsed laser deposition (PLD) in a high vacuum chamber [21–22]. a-Al_2_O_3_ and the metals are deposited by PLD using a KrF excimer laser at 248 nm with a pulse duration of 25 ns at a repetition rate of 5 Hz. Substrates were commercial transmission electron microscopy (TEM) grids on which an amorphous carbon layer with a thickness of 10 nm was deposited. On the top of the amorphous carbon, a 3 nm layer of a-Al_2_O_3_ was deposited. Then, cobalt and platinum were alternatively deposited using pure Co and Pt targets irradiated with an energy density of 4.4 J/cm^2^ in order to obtain Co_50_Pt_50_ NPs. The crystalline structure of as-grown NPs can be controlled with the substrate temperature [21–22]. Two samples with a nominal thickness of 2.5 nm were prepared, with a substrate temperature of 550 °C and 650 °C, leading to the formation of a disordered face centered cubic (FCC) and L1_0_ ordered structures, respectively. On both samples, a 3 nm-thick layer of a-Al_2_O_3_ was deposited over the NPs to protect them from air oxidation.

**Figure 1:**
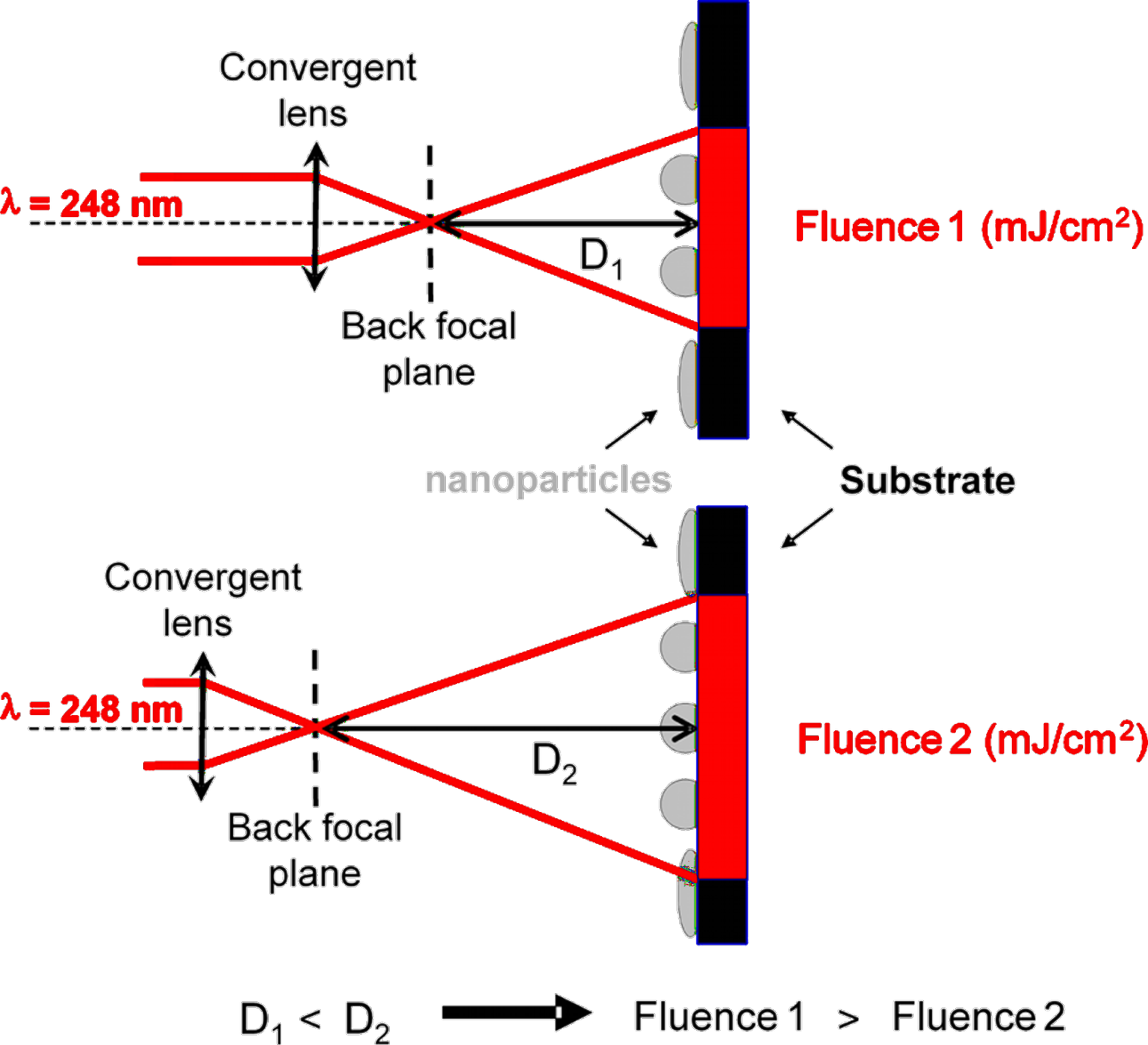
Schematic representation of the experimental setup: the sample is placed behind the back focal plane of a convergent lens. The fluence in the substrate plane is then fixed by the distance D between the sample and the back focal plane of the lens. The longer is D, the lower is the fluence on the substrate.

After the synthesis, the sample was irradiated by using the same laser as the one used for the PLD experiment. A pulse frequency of 1 Hz was used and the laser energy was chosen well below the ablation threshold of CoPt and Al_2_O_3_ in order to avoid the vaporization of the sample. For that purpose, a focusing lens is placed between the laser and the sample. The experimental setup is schematically illustrated in Figure 1. The energy density on the sample is controlled by the distance D between the back focal plane of the lens and the sample. In our experiment, samples were irradiated with a fluence of 47 mJ/cm^2^. The evolution of the NPs size and shape was studied by TEM. TEM experiments were carried out on a JEM-2010F field-emission electron microscope operating at 200 kV and equipped together with a high-resolution pole piece and a PGT energy dispersive X-Ray (EDX) analyser.

**Figure 2:**
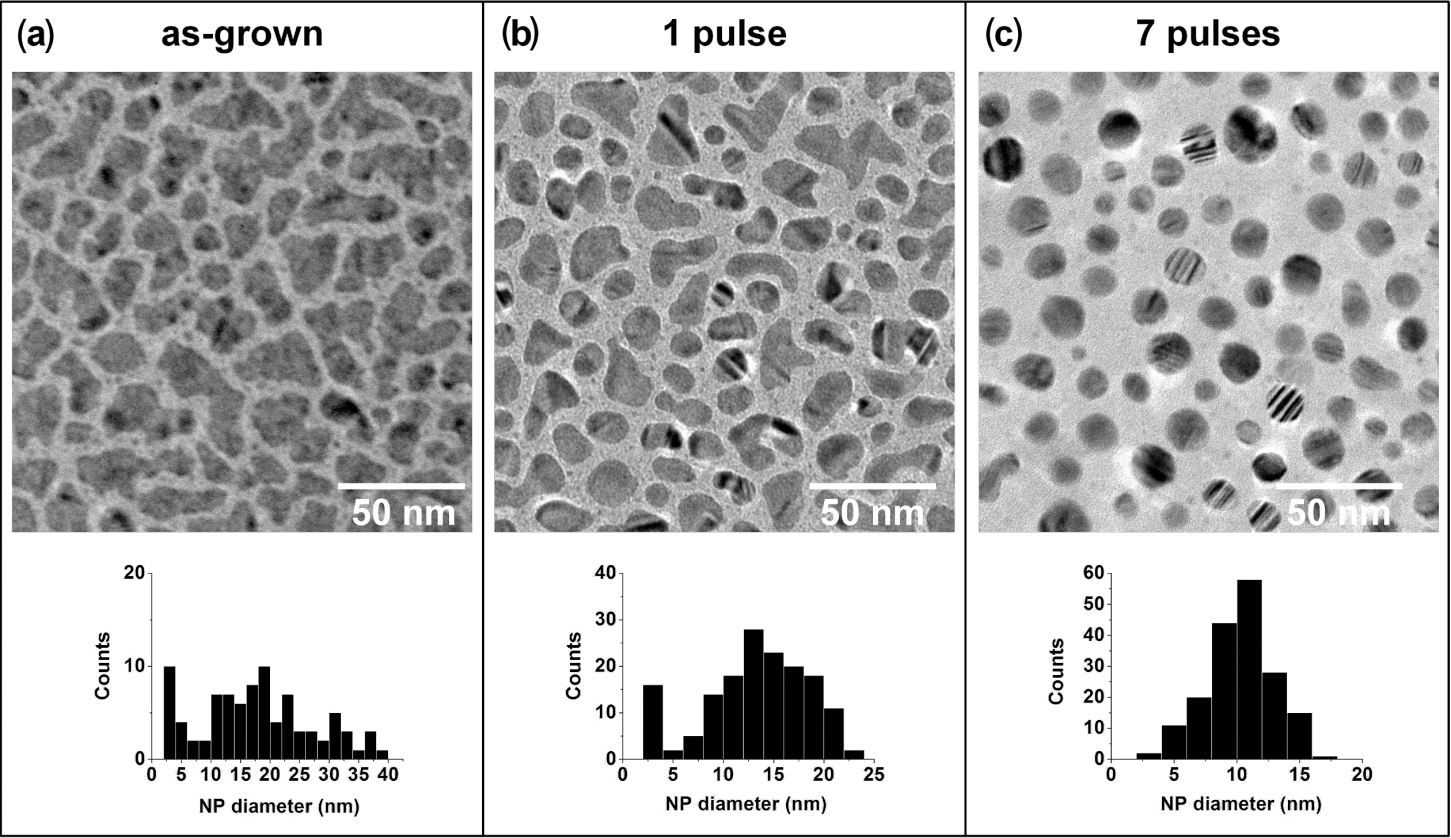
Evolution of the CoPt NPs size and shape as a function of the number of laser pulses. TEM images and the corresponding NPs size dispersion, (a) before laser irradiation, (b) after 1 laser pulse, (c) after 7 laser pulses.

The morphology of as-grown CoPt NPs is shown in Figure 2a. NPs have irregular shapes elongated in the substrate plane due to coalescence processes during the synthesis. The morphological changes induced by the laser irradiation as a function of the number of laser pulses is presented in Figure 2b and Figure 2c. After the first pulse, we can already observe a partial reshaping of the NPs towards rounded shapes and smooth surfaces, but a significant number of elongated NPs remains. After 7 laser pulses, the shape and the size distribution of bimetallic NPs have completely changed. First of all, the morphology of the particles evolves from a flat to spherical shape, as indicated by the higher intensity levels of the NPs in Figure 2c. At the same time, the mean size, the polydispersity, and the coverage ratio of the NPs decrease (Table 1), changing the broad size dispersion of as-grown NPs into a Gaussian distribution (Figure 2). This technique allows the fabrication of 10 to 15 nm size NPs with a polydispersity as low as 20%. In good agreement with previous studies on monometallic NPs [23–24], we have shown that similar effects are obtained with CoPt thin films near the percolation threshold, indicating that the morphological transformations does not depend on the as-grown film morphology. If flash laser annealing experiments always result in spherical and monodisperse NPs, the nominal thickness of the as-grown film can be used to control the final size of the irradiated NPs.

**Table 1:** Evolution of the NPs mean size, polydispersity, and coverage ratio as a function of the number of laser pulses.

	As-grown	1 pulse	7 pulses

Mean size	16.6 nm	13.4 nm	10.3 nm
Polydispersity	61%	38%	25%
Coverage ratio	60%	53%	32%

NPs composition was measured by EDX analysis. Despite the complete change of the NP morphology, their composition was found to be Co_50_Pt_50_ (±2 at. %) before and after irradiation experiments. Therefore flash laser annealing does not influence the composition of bimetallic cluster which is a *sine qua non* condition for the use of this technique on magnetic alloy NPs. The conservation of NPs composition indicates that if metal atoms evaporate from CoPt NPs thin films during flash laser annealing, the evaporation rates of Co and Pt atoms are similar. This property of alloys is sometimes exploited for controlling the composition of NPs synthesized by PLD, since the irradiation of alloy targets often leads to the formation of NPs with the same stoichiometry as the target [25–26].

The laser energy can be absorbed either by the NPs or by the substrate since both materials absorb at the laser wavelength. For the NPs, this absorption results in the increase of their temperature and induces desorption of Co and Pt atoms. The absorption cross section of the UV radiation varies as d^3^, where d is the diameter of the NPs [13,27]. Desorption phenomena are then more effective on the biggest particles leading to the reduction of the particle size and polydispersity (Table 1). This process can be described, as an “inverse” Ostwald ripening [28], since energetic factors cause small NPs to grow, drawing materials from the bigger clusters, which shrink. In addition, NPs polydispersity is also reduced by the disappearance of the sub-3 nm clusters, which are unstable under laser irradiation because of their lower melting temperature [29]. After a few laser pulses, all the particles are large enough to remain stable under laser irradiation, and their narrow size dispersion tends to equilibrate the laser-induced fluxes of atoms between the clusters.

In parallel, the temperature increase due to the laser intensity induces a solid-liquid transition of the alloy leading to a complete reshaping of the particles. This solid-liquid transition is demonstrated by the rounded shape of the particles similar to small water droplets on clean glass substrate. The formation of twin boundaries observed on Figure 2c, is characteristic of rapid solidification processes following NPs melting. These laser-induced phenomena tend to reduce the surface energy of the NPs [19] and spherical shape is the energetically favourable configuration. Evidence of NPs melting has been also reported for irradiated Au NPs [15,30].

**Figure 3:**
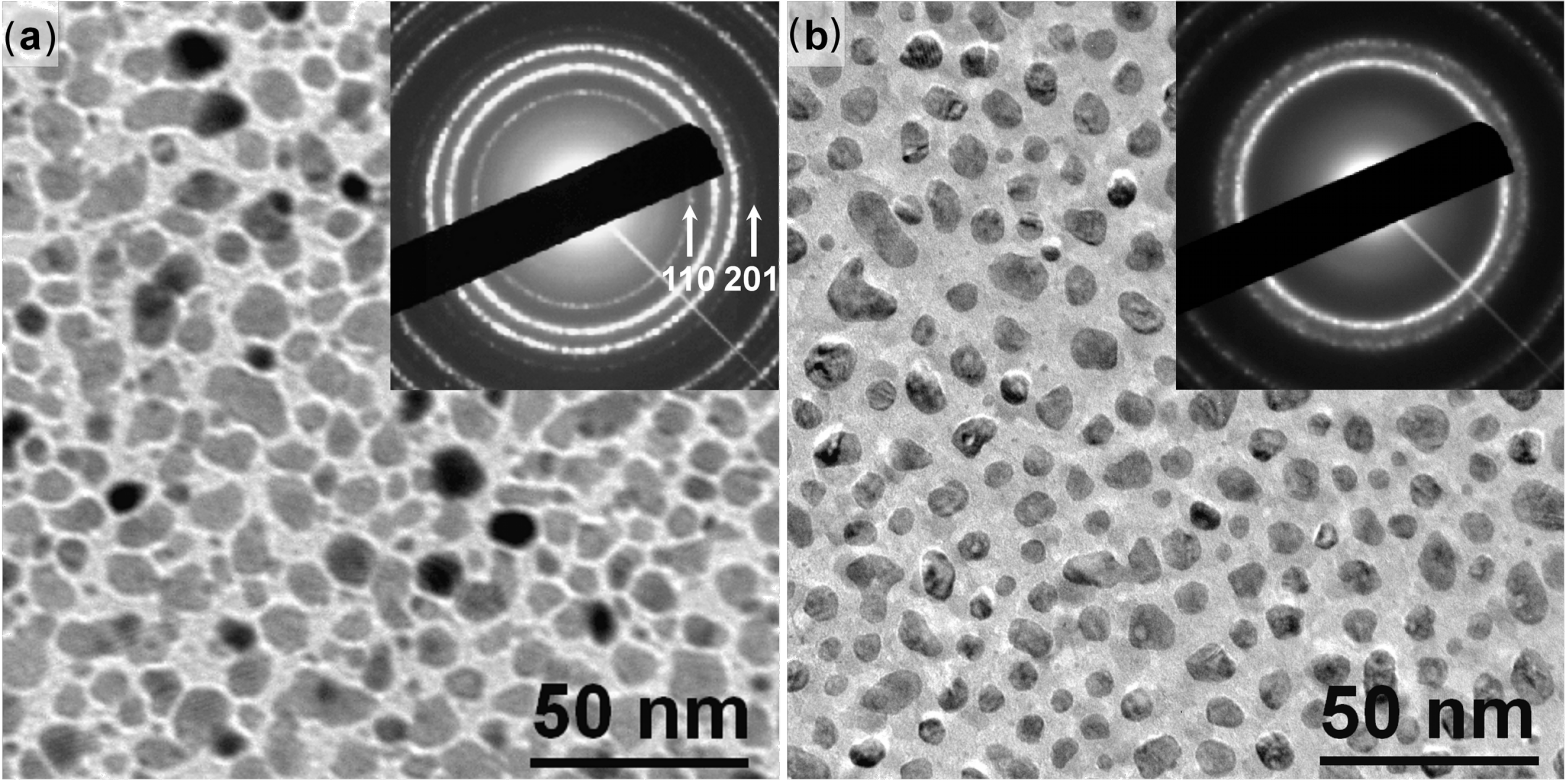
Evolution of the CoPt NPs size, shape, and crystalline structure during flash laser annealing. TEM images and the corresponding diffraction pattern (inset), (a) as-grown CoPt NPs in the L1_0_ ordered structure, (b) after 3 laser pulses, CoPt NPs in the FCC disordered structure.

Bulk CoPt alloy has a phase transition at 825 °C between the L1_0_ ordered structure at low temperature and the disordered FCC structure at high temperature. As previously reported [9], this phase transition temperature decreases with particle size; however, such a size effect only occurs in sub-3 nm CoPt NPs. It can then be considered that the phase transition temperature for NPs larger than 10 nm is similar to the bulk phase transition temperature. Figure 3 shows that flash laser annealing experiments performed on L1_0_ ordered CoPt NPs result in FCC clusters. This phase transformation is demonstrated by the disappearance of the 110 and 201 superstructure reflections, characteristic of chemically ordered structures, on the diffraction pattern of the NPs (Figure 3b). This result proves that the temperature inside the NPs is at least higher than 825 °C. Moreover, this disordering is similar to a quenching of the NPs from a high temperature phase and demonstrates the very fast thermalisation of the NPs, during which the substrate probably acts as a heat sink.

Of course, FCC disordered NPs are not usable for information storage applications because of their superparamagnetic state. However, we have previously reported [21] that between 600 °C and 700 °C, the temperature is high enough to transform FCC NPs into chemically ordered NPs and low enough to prevent NPs coalescence. Using classical annealing procedures L1_0_ ordered NPs can then be obtained without changing their shape.

In conclusion, flash laser annealing is a method of choice to fabricate 10 to 15 nm size magnetic alloy NPs with spherical shape and low polydispersity (~20%). Indeed, in this range of size, conventional chemical and physical syntheses do not allow the fabrication of NPs with such narrow size dispersion. This technique gives an unprecedented opportunity to control the size and the shape of bimetallic NPs without changing their composition. It can also be used to produce organized CoPt or any bimetallic NPs on a substrate, by using an accurate patterning of the light field intensity designed by masks or gratings lithography [14,31].
